# Ovarian Teratoma in a Nonverbal Adult With Rett Syndrome

**DOI:** 10.7759/cureus.114672

**Published:** 2026-08-17

**Authors:** Aleeza Mae V Garcia, Danielle L Hudman, Paul McGowan, Brandy Ree

**Affiliations:** 1 Medical School, Arkansas College of Osteopathic Medicine, Fort Smith, USA; 2 Pathology, Arkansas College of Osteopathic Medicine, Fort Smith, USA; 3 Molecular and Cellular Biology, Arkansas College of Osteopathic Medicine, Fort Smith, USA

**Keywords:** benign neoplasm, cancer, cystic teratoma, germ cell tumor, mecp2, ovarian teratoma, rett syndrome, tumor

## Abstract

Mature cystic ovarian teratomas, also called dermoid cysts, are the most common ovarian neoplasms in women, especially those of reproductive age. While the general clinical approach and treatment of teratomas do not differ in a patient with a concomitant diagnosis of Rett syndrome, the presentation, surgical preparation, and clinical course can be influenced by the pathophysiology of the condition. We report the case of a 35-year-old nonverbal woman with Rett syndrome presenting with symptoms of abdominal pain, constipation, abdominal distension, nausea, and vomiting. Computed tomography of the abdomen and pelvis revealed a mass in the left lower quadrant, originating from the left adnexa. During an exploratory laparotomy, a large cystic mass was identified in the region of the left adnexa with associated adnexal torsion, and the patient underwent a left salpingo-oophorectomy. Microscopic examination of the adnexal mass confirmed a diagnosis of mature ovarian cystic teratoma. This report adds to the limited literature on gynecologic pathology in Rett syndrome.

## Introduction

Rett syndrome is a rare neurodevelopmental disorder that typically affects young females and is characterized by an initial period of normal development followed by neurodevelopmental regression within the first year of life, leading to hallmark features, including gait abnormalities, loss of purposeful hand movements, stereotypic hand-wringing behaviors, and loss of acquired language skills [1]. The syndrome is often a result of a mutation in methyl-CpG-binding protein 2 (MECP2), a nuclear protein that binds to sequences of methylated cytosine in DNA, permitting regulation of chromatin structure [1]. In humans, MECP2 is found in various cell types but is concentrated in the brain to perform essential roles in neuronal differentiation during early embryonic development, neuronal maturation, circuit formation, and maintenance of neuronal function [2].

Benign mature cystic teratomas are the most common ovarian germ cell tumors and are responsible for 20% of all ovarian neoplasms [3]. They are often asymptomatic until complications from mass effect arise, which occur in about 16% of patients with ovarian teratomas [3]. Although cystic teratomas are benign, patients who present with symptomatic mature teratomas are recommended to undergo laparoscopic cystectomy or, in the case of those larger than five centimeters, an oophorectomy [4]. As patients with Rett syndrome now routinely survive into adulthood, the initial symptoms of medical conditions, such as the development of gynecologic neoplasms like ovarian teratomas, may present with less typical features due to their speech and motor disabilities. Here we present a 35-year-old woman with Rett syndrome who presented to the hospital with symptoms consistent with small bowel obstruction and was later found to have a mature cystic ovarian teratoma. To our knowledge, this is the first report of the rare coexistence of Rett syndrome and mature cystic ovarian teratoma in the literature.

This article was previously presented at the first annual Conway Regional Health System Research Symposium on October 10, 2025.

## Case presentation

**Figure 1:**
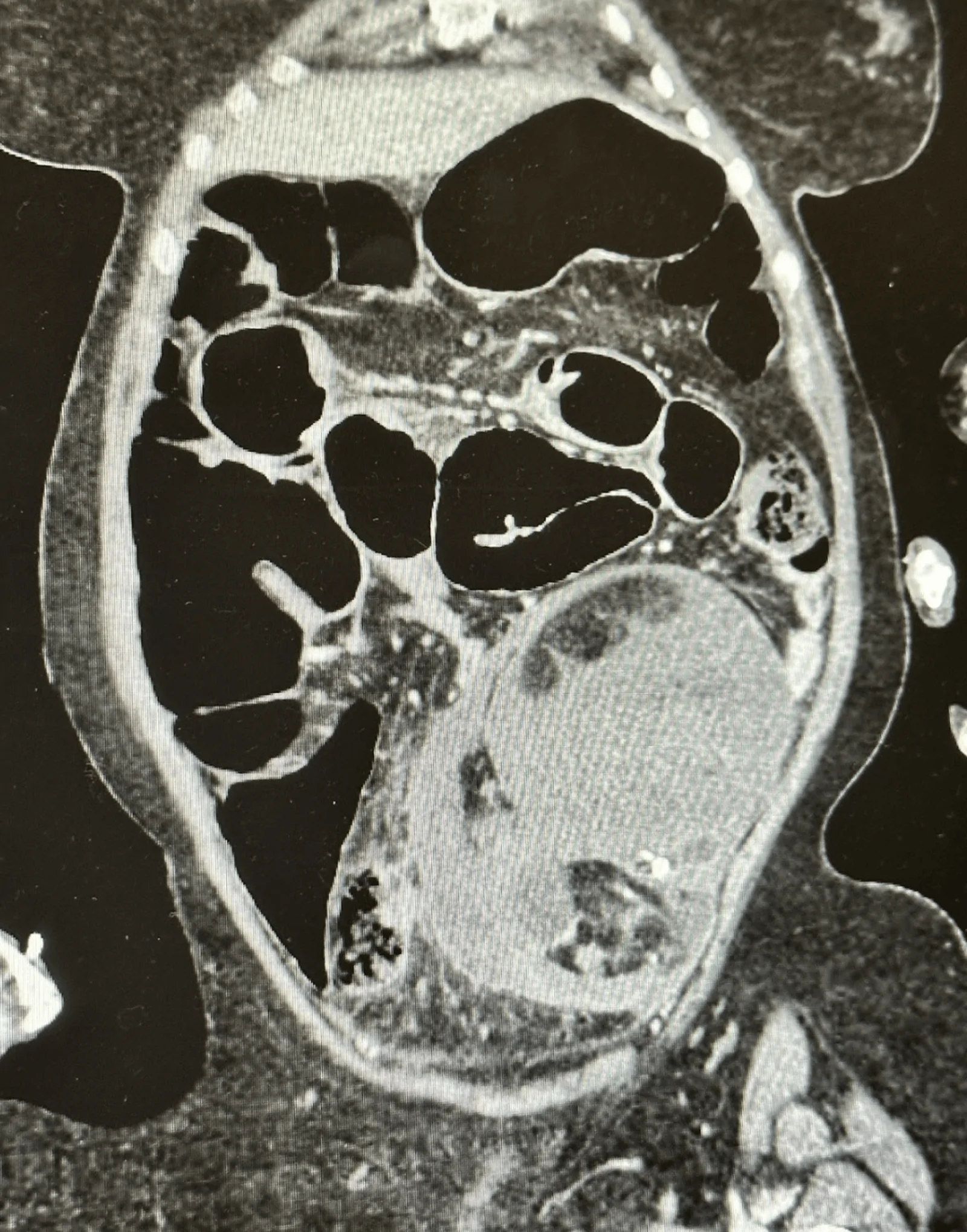
Computed tomography of abdomen and pelvis revealed a 14.9 cm mixed density mass in the left lower abdomen/left pelvis, possible small complex fluid collection along the medial margin of the mass, and distended stomach with proximal mid-small bowel with scattered air fluid levels.

The patient is a 35-year-old nonverbal woman with a history of Rett syndrome and a well-controlled seizure disorder who presented to the emergency department (ED) after three days of abdominal bloating, vomiting, cough, and congestion. On arrival, the patient was afebrile, mildly hypertensive, tachycardic, and tachypneic (Table 1). Her physical examination was remarkable for a distended abdomen with high-pitched bowel sounds and apparent tenderness in the left lower quadrant, though assessment was limited by her inability to communicate. A palpable mass was appreciated in the same region. Laboratory results were remarkable for leukocytosis at 31.57, hyponatremia at 130, and elevated lactic acid at 3.0. Beta-human chorionic gonadotropin (β-hCG) was negative. Her chest X-ray demonstrated diffuse interstitial opacities, and computed tomography of the abdomen and pelvis revealed a 14.9 cm mixed-density mass in the left lower abdomen/pelvis, likely arising from the left adnexa, with a small complex fluid collection medially (Figure 1).

**Table 1:** Timeline of clinical course.

Day	Parameter/Finding	Result/Details	Intervention
Hospital Day 1	Heart rate	125 beats per minute	Work up for sepsis: obtain blood cultures, initiate empiric antibiotics, and intravenous fluid resuscitation. Work up for small bowel obstruction: bowel rest and nasogastric tube decompression.
	Blood pressure	139/91	
	Respiratory rate	40 per minute	
	Oxygen saturation	94%	
	White blood cell	31.57 × 10³/µL	
	Sodium	130 mEq/L	
	Lactic acid	3.0 mmol/L	
	Chest X-ray	Diffused interstitial opacities, possible early infection	
	CT Abdomen and Pelvis	14.9 cm mixed-density mass in the left lower abdomen/pelvis, likely arising from the left adnexa, with a small complex fluid collection medially. There is gastric and proximal small bowel dilation with scattered air-fluid levels and a transition point near the superior margin of the pelvic mass.	
Hospital Day 2	Clinical course	Patient has two bowel movements	Continue to monitor
Hospital Day 3	Small bowel follow-through	Gastrografin contrast does not progress into distal small bowel or colon, concerning for a small bowel obstruction.	The patient is scheduled for non-emergent surgery for the following day.
Hospital Day 4	Clinical course	The patient is taken to the operating room.	Exploratory laparotomy, excision of left pelvic mass, and left salpingo-oophorectomy
Hospital Day 5 (Postoperative Day 1)	Respiratory panel	Rhinovirus and enterovirus positive	Empiric antibiotics are discontinued. Proceed with supportive care for viral pneumonia.
	Blood cultures	Negative, without growth	
	Small bowel follow-through	Progression of gastrografin contrast to the colon. No small bowel obstruction visualized.	
Hospital Day 7 (Postoperative Day 3)	Clinical course	Patient begins to tolerate a clear liquid diet. Patient has one normal bowel movement.	Nasogastric tube is discontinued
Hospital Day 14 (Postoperative Day 10)	Clinical course	In the interval, the patient was gradually advanced to full liquid and regular diet, respectively; their respiratory infection has cleared; and clinical lab values have improved.	Patient is discharged home

**Figure 2:**
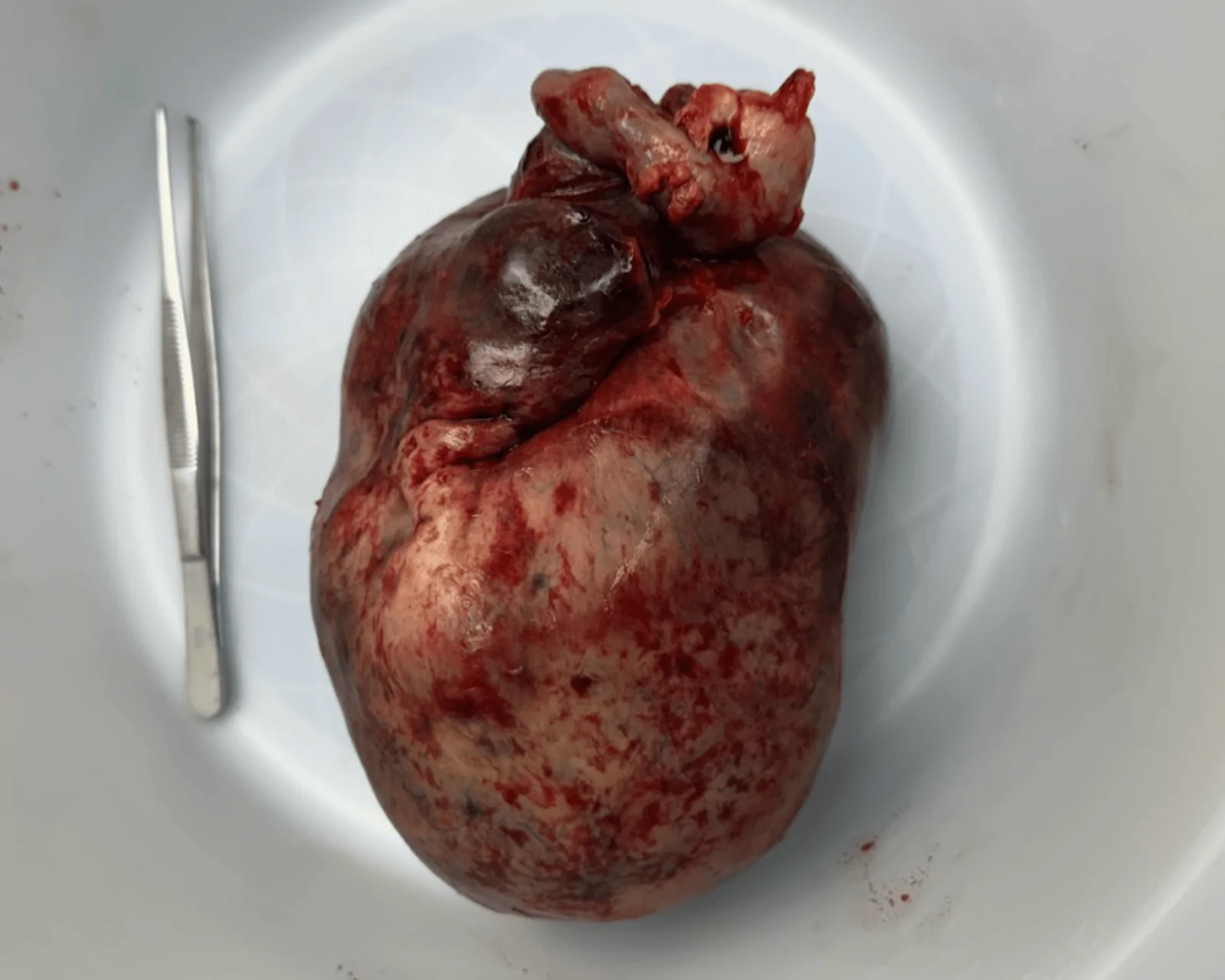
Gross appearance of the large, left adnexal tumor.

There was also notable gastric and proximal small bowel dilation with scattered air-fluid levels and a small bowel transition point near the superior margin of the pelvic mass. An initial impression of small bowel obstruction (SBO) and sepsis secondary to a respiratory infection was made, and the patient was subsequently started on broad-spectrum antibiotics and nonoperative management of SBO, which included bowel rest, nasogastric (NG) tube decompression, and intravenous fluids. Although the patient had two bowel movements on hospital day 2, a small bowel follow-through was performed on hospital day 3, which demonstrated progression of Gastrografin into the mid-small bowel but none into the distal small bowel or colon. Due to failure of nonoperative management of SBO, the decision was made for exploratory laparotomy on hospital day 4. Intraoperatively, a pelvic mass was discovered involving an enlarged left ovary with at least 360° torsion of the uterine tubes. The uterus and right adnexa appeared normal. Due to its complex involvement with the left ovary and left uterine tubes, the pelvic mass was removed via salpingo-oophorectomy (Figure 2).

On hospital day 5 (postoperative day 1), a repeat small bowel follow-through showed the progression of contrast throughout the small bowel and colon. Additionally, the patient’s respiratory panel returned positive for rhinovirus and enterovirus. At this time, the patient’s blood cultures remained persistently negative, and empiric antibiotics were discontinued with the decision to manage the diagnosis of viral pneumonia with supportive care. On hospital day 7 (postoperative day 3), the patient began to tolerate a clear liquid diet and was able to have one small bowel movement, and her NG tube was subsequently removed. From this standpoint, it took an additional six days for the patient to fully tolerate a regular diet. In the interval, the pathologic examination of the excised pelvic mass had resulted, reporting sections of the left ovary with foci of skin with adnexa, adipose tissue, and cartilage, which confirmed the diagnosis of ovarian cystic teratoma with associated diffuse hemorrhagic necrosis and fallopian tube hemorrhagic necrosis. Upon resolution of her respiratory infection, improvement of clinical lab values, and tolerance of a regular diet, the patient was discharged home on hospital day 14 (postoperative day 10). 

## Discussion

Rett syndrome 

Rett syndrome is estimated to have a prevalence of about 1 in 100,000 live female births [2]. It is rarely diagnosed in males, likely due to the potential lethal effects of a single X chromosome with a MECP2 mutation, resulting in severe neonatal encephalopathy [1]. Rett syndrome most often results from mutations in the MECP2 gene located on Xq28 [1]. It tends to manifest around 6-18 months of age after a period of normal early development, and its presentation often includes the loss of purposeful hand movements, development of repetitive hand movements, loss of spoken language, and gait abnormalities [1]. This was the case for our patient. Others may also present with deceleration of head growth, seizures, autism-like behaviors, and irregular breathing. MECP2 mutations have been well established to manifest in a spectrum of neurodevelopmental disorders such as Rett syndrome, autism spectrum disorder, neonatal encephalopathy, and X-linked intellectual disability [5].

More recently, studies have speculated on the potential role of MECP2 in the development of various human cancers, such as breast, colorectal, pancreatic, gastric, hepatocellular, and prostate cancer [6]. Higher levels of MECP2 mRNA have been found in human neoplastic breast tissue when compared to non-neoplastic breast tissue [7]. Additionally, increased levels of MECP2 gene transcription have been described in well-differentiated adenocarcinoma and mucinous adenoma-containing tissues [8]. When MECP2 expression is inhibited, there appears to be a reduction in prostate cancer progression, while higher levels of expression are associated with greater cell proliferation [9]. While these studies suggest that the overexpression of MECP2 may be associated with tumor formation, it is interesting to note the development of an ovarian teratoma in a patient with Rett syndrome, which often results from MECP2 loss-of-function mutations. In the literature, very few Rett syndrome patients have been reported to present with various types of malignancies. These include tuberous sclerosis [10], subungual malignant melanoma [11], squamous cell carcinoma of the trachea and lung [12], colon cancer [5], breast cancer [5], prostate cancer [5], and osteosarcoma [13]. One article reported a case of serous ovarian borderline tumor that presented with villous adenoma with high-grade dysplasia of the colon [14]. It raises the question of whether any kind of MECP2 dysfunction, whether due to overexpression or underexpression, could influence pathways of cellular differentiation and potentially contribute to tumor formation; however, given how rare Rett syndrome is and the even fewer case reports describing its coexistence with tumor development, it is rather likely that the incidence of ovarian teratoma in our patient with Rett syndrome may be coincidental.

Ovarian teratoma 

Mature cystic ovarian teratomas are benign neoplasms that are formed by two or three germ cell layers (ectoderm, endoderm, mesoderm) [3]. They account for 20% of ovarian neoplasms but are often asymptomatic until their size begins to affect surrounding anatomical structures, typically between the ages of 20 and 40 years [3]. Other subtypes of ovarian teratomas include immature (malignant), malignant secondary to intracystic components, and monodermal teratomas [3]. Depending on the dermoid constitution, presentations may include hyperthyroidism due to ectopic thyroid tissue (struma ovarii) [15] or paraneoplastic anti-NMDA receptor encephalitis [4]. Parasitic dermoids, resulting from autoamputation and reimplantation onto the omentum, can present with abdominal pain due to ischemia and adhesion formation [4]. Some proposed risk factors for teratoma development are late menarche with menstrual irregularities, alcohol use disorder, history of a prior teratoma, lower parity, infertility, and exercise during adolescence leading to anovulatory cycles [4]. Although ovarian teratomas are not known to be associated with specific elevations in tumor markers, they are instead most often diagnosed based on their characteristic signs on several imaging modalities such as computed tomography, ultrasound, and radiography [3].

When considering an ovarian mass in the context of Rett syndrome, several caveats must be addressed. Our patient, who did not have any of the risk factors mentioned above, may have had this teratoma for an unknown period of time prior to its diagnosis. Its discovery at the size of 14.9 cm, upon presentation to the hospital with symptoms suggestive of bowel obstruction, was likely influenced by her inability to verbalize or physically demonstrate discomfort from the growing mass. Due to her history of Rett syndrome, our patient has a severe motor disability and is chronically in a forward-flexed position, which can also make it difficult to visualize the abnormal presentation of her abdomen. Bowel irregularities are also common in patients with Rett syndrome [1]. Because of this, it can be especially difficult for caretakers to suspect tumor growth as a cause of obstruction or a contributor to our patient’s history of constipation until symptoms become more severe, which ultimately prompted our patient’s presentation in the ED. Due to the classic repetitive hand movements and the 60% risk of seizure disorder, certain imaging modalities may be limited by motion artifacts. Although our patient, who was unsedated, tolerated undergoing a CT scan, any movement during the process may have limited visualization of adnexal torsion. Roughly 12% of mature teratomas are bilateral, so it is important to carefully evaluate the contralateral adnexa [3]. In our case, the patient’s contralateral adnexa did not present with any abnormal features on CT imaging or during operative evaluation. The patient’s tenderness in the left lower quadrant while in the ED may have been due to adnexal torsion that was not obvious on her initial CT scan, but because the initial impression of small bowel obstruction was made, the patient was not taken into the operating room until failure of nonoperative SBO management. Her apparent adnexal torsion was not discovered until intraoperative examination during her exploratory laparotomy and was eventually confirmed by the hemorrhage and necrosis noted on the pathology report. Postoperatively, an ileus or delays in return of spontaneous bowel function may also be exacerbated by the patient’s limited mobility and, in our patient's case, potentially contributed to her slow, gradual tolerance of a regular diet.

Limitations

This case report describes the presentation of a patient with Rett syndrome who was found to have a pelvic mass consistent with the diagnosis of ovarian cystic teratoma; however, the case report has several limitations. Certain clinical lab values, such as alpha-fetoprotein (AFP) and lactate dehydrogenase (LDH), were not obtained, which limited a more comprehensive evaluation of the patient’s tumor subtype. Analysis for CA-125 or CA 19-9 tumor markers was also not performed. Additionally, while the excised pelvic cyst was sent for pathologic examination, we were unable to include the histopathologic images in this report due to a lack of correspondence from the hospital’s pathology department despite numerous requests. Moreover, as this is a single-case report, it is not possible to establish causality or determine the effectiveness of the management approach. However, the detailed clinical description and outcome can provide useful information for clinicians encountering similar presentations.

## Conclusions

To our knowledge, this is the first report of a patient with Rett syndrome presenting with mature cystic ovarian teratoma in the literature. Disordered MECP2 expression has been implicated in the progression of certain malignancies, whereas reduced expression is sometimes associated with diminished tumor growth. Interestingly, the literature revealed multiple cases involving various tumors that presented in the setting of Rett syndrome, a neurodevelopmental disorder that often results from MECP2 loss-of-function mutations. Due to the rarity of Rett syndrome and lack of reports that describe Rett syndrome patients with gynecologic abnormalities, it would be difficult to speculate whether Rett syndrome or its underlying MECP2 mutation plays a role in predisposing patients to any level of dysregulated cell growth or formation of benign growths such as ovarian teratomas. This rare presentation may be purely coincidental and would require larger cohort studies to suggest any causality.

Given the ubiquitous nature of ovarian teratomas and MECP2 emerging as a potential oncogene, it is important to consider a growing mass resulting in pathologic effects as a culprit for new-onset symptoms presenting alongside Rett syndrome. This case underscores the importance of a comprehensive clinical approach, particularly in patients with impaired communication and mobility, such as those with Rett syndrome. Careful evaluation and thoughtful perioperative planning are essential to optimize outcomes and support a smooth recovery.
